# Allocation Patterns and Strategies of Carbon, Nitrogen, and Phosphorus Densities in Three Typical Desert Plants

**DOI:** 10.3390/plants14111595

**Published:** 2025-05-23

**Authors:** Guangxing Zhao, Akash Tariq, Zhaobin Mu, Zhihao Zhang, Corina Graciano, Mengfei Cong, Xinping Dong, Jordi Sardans, Dhafer A. Al-Bakre, Josep Penuelas, Fanjiang Zeng

**Affiliations:** 1 Xinjiang Key Laboratory of Desert Plant Roots Ecology and Vegetation Restoration, Xinjiang Institute of Ecology and Geography, Chinese Academy of Sciences, Urumqi 830011, China; zhaoguangxing22@mails.ucas.ac.cn (G.Z.); muzhaobin@ms.xjb.ac.cn (Z.M.); zhangzh@ms.xjb.ac.cn (Z.Z.); congmengfei95@163.com (M.C.); dongxinping23@mails.ucas.ac.cn (X.D.); 2 Cele National Station of Observation and Research for Desert-Grassland Ecosystems, Cele 848300, China; 3 State Key Laboratory of Ecological Safety and Sustainable Development in Arid Lands, Xinjiang Institute of Ecology and Geography, Chinese Academy of Sciences, Urumqi 830011, China; 4 University of Chinese Academy of Sciences, Beijing 100049, China; 5 Consejo Superior de Investigaciones Cientificas (CSIC) Global Ecology Unit, CREAF-CSIC-UAB, Bellaterra, 08193 Barcelona, Catalonia, Spain; j.sardans@creaf.uab.cat (J.S.); josep.penuelas@uab.cat (J.P.); 6 Centre for Ecological Research and Forestry Applications (CREAF), Cerdanyola del Vallès, 08193 Barcelona, Catalonia, Spain; 7 Instituto de Fisiología Vegetal, Consejo Nacional de Investigaciones Científicas y Técnicas, Universidad Nacional de La Plata, Buenos Aires B1406, Argentina; corinagraciano@gmail.com; 8 College of Resource and Environment Sciences, Xinjiang University, Urumqi 830046, China; 9 Department of Biology, College of Science, University of Tabuk, Tabuk 71491, Saudi Arabia; dalbakre@ut.edu.sa

**Keywords:** nutrient-use strategy, adaptive differentiation, biomass allocation, carbon–nitrogen–phosphorous densities

## Abstract

The densities of carbon, nitrogen, and phosphorus (C-N-P) reflect the adaptation and response of desert plants to hyper-arid environments. However, the allocation strategies for biomass and C-N-P densities among various plant life forms remain poorly understood. This study involved the collection of samples representing both aboveground and belowground biomass (to depths of 200 cm) from three desert plant species—both herbaceous and shrubby—and evaluating their C-N-P densities. The investigation focused on the distribution strategies and drivers influencing total C-N-P densities within the plant–soil system. The results indicated that the biomass of the shrub *Tamarix ramosissima* (8.88 ± 1.22 kg m^−2^) was significantly greater than that of the herbaceous plants *Alhagi sparsifolia* (0.96 ± 0.15 kg m^−2^) and *Karelinia caspia* (0.72 ± 0.09 kg m^−2^). The total C density among the three species was observed as follows: *T. ramosissima* (9.26 ± 0.99 kg m^−2^) > *A. sparsifolia* (6.21 ± 0.85 kg m^−2^) > *K. caspia* (6.18 ± 1.12 kg m^−2^). Notably, no significant differences were detected in the total N and P densities across the species. Additionally, for *A. sparsifolia* and *K. caspia*, the roots exhibited greater biomass and C-N-P densities. Further analysis revealed that soil pools accounted for 56.34–95.10% of total C density, 90.39–98.63% of total N density, and 99.86–99.97% of total P density in the plant–soil system. The order of total C-N-P densities was established as C > P > N, decoupling total P density from other environmental factors. Total C and N densities in the three plant species were predominantly influenced by soil physicochemical properties, with biotic factors and microbial biomass playing secondary roles. This study improves the understanding of C-N-P densities strategies of dominant vegetation for restoration and sustainable management in hyper-arid deserts.

## 1. Introduction

Desert ecosystems, encompassing approximately 20% of the world’s terrestrial land area, exhibit lower biodiversity and productivity. Nevertheless, desert plants play a vital role in environmental stabilization, through windbreak effects, sand fixation, soil conservation, and enhancing biodiversity [1]. Adapted to extreme aridity, high temperatures, limited water availability, and nutrient deficiencies, desert plants utilize unique physiological adaptations to thrive [2]. These adaptations enable desert vegetation to stabilize soils, retain essential nutrients (particularly nitrogen [N] and phosphorus [P]), maintain soil organic matter (SOM), and significantly reduce soil erosion, forming critical plant–soil nutrient pools [3,4]. Carbon (C) forms the fundamental basis of all organic compounds and is crucial for all living organisms [4,5]. Through photosynthesis, plants mediate climate regulation, soil structure, and nutrient cycling [6]. N and P serve as essential components of amino acids and nucleic acids, critical for plant growth, community stability, and ecosystem development [7,8]. Biomass C-N-P pools correlate with plant growth and productivity, while soil N and P pools are vital for plant nutrition; both are linked to soil C pools. Desert plants facilitate soil nutrient cycling and ecological functions by absorbing, mobilizing, and translocating C, N, and P through their root systems [9,10].Their efficiency in managing these nutrient processes underscores their crucial role in sustaining ecosystem balance in some of the world’s harshest environments.

Plant growth, reproduction, and life cycle are fundamentally linked to C-N-P accumulation and cycling [11]. Nutrient allocation strategies in plants principally govern the distribution of nutrients among organs (roots, stems, leaves) and regulate intra-organ C-N-P partitioning [12]. Various environmental factors, such as drought, high temperatures, and nutrient deficiencies, influence different plant organs’ elemental requirements [13]. The allocation of nutrients reflects the relative investment in proteins or RNAs across different organs [14], and evolutionary trade-offs between plant adaptation to the environment and resource acquisition [15,16]. In response to persistent drought and thermal stress, desert plants have evolved specialized adaptive strategies through evolutionary time. Examples of these adaptations include deeper root systems, reduced transpiration rates, and the development of thick leaves, thorns, or assimilative branches [17,18].

Herbaceous plants and shrubs often occupy distinct ecological niches due to resource competition, which leads to diverse strategies for nutrient uptake and storage [19]. Research suggests that herbaceous species may require more nutrients to cope with drought, while woody plants exhibit greater drought tolerance and require fewer nutrients [20]. Differences in C-N-P concentrations among plants are common, with herbaceous plants typically displaying lower nitrogen to phosphorus (N:P) ratios [21]. In desert ecosystems, the belowground nutrient pool—including roots and soil—often assumes a dominant role [22]. A correlation frequently exists between plant nutrient levels and soil nutrients, as approximately 88% of the N required by plants is derived from soil nitrogen globally [23]. Moreover, interaction and coupling processes among elements can significantly affect material cycling within ecosystems. For instance, both N and P regulate microbial activity and ecosystem productivity, which in turn influences litter and root decomposition quality and quantity, ultimately affecting C cycling [24,25]. Changes in soil C-N-P densities are typically influenced by a combination of arid environmental factors; however, in specific natural ecosystems, soil properties and vegetation may impact total C-N-P pools more strongly than climatic factors [26]. In arid regions, challenges such as limited nutrient availability and high temperatures are prevalent, complicating the nutrient release and cycling processes within plant–soil interactions [27,28].

Ecosystems demonstrate distinct allocation strategies for soil C-N-P densities, which are fundamentally regulated by plant physiological and growth characteristics [29,30]. Numerous studies have focused on quantifying and allocating C-N-P densities in forest and grassland ecosystems, primarily at the 0–60 cm soil depth [31,32,33]. Nevertheless, research on the distribution strategies of C-N-P densities in deeper soil layers (beyond 60 cm) is notably lacking. While recent studies prioritize quantifying plant–soil C density ratios [34], concomitant analyses of N and P pools—essential for predicting long-term C sink sustainability—are frequently omitted. The complexity and high costs associated with sampling have limited quantitative analysis of C-N-P densities and allocation mechanisms across desert plant–soil systems.

Thus, this study selected three species representing different types of natural desert plants: two herbaceous species, *Alhagi sparsifolia* Shap. (Fabaceae), *Karelinia caspia* Pall. (Compositae), and one shrub, *Tamarix ramosissima* Ledeb from the transition zone between desert and oasis environments. The specific objectives of the study included: (1) How different plant life forms (herbaceous vs. shrub) allocate C-N-P resources; (2) Investigating the distribution ratio of C-N-P densities within the plant–soil system; and (3) Identifying the primary driving factors influencing total C-N-P densities. The proposed hypotheses were as follows: (1) Desert herbaceous plants allocate a larger proportion of their C-N-P densities to their roots than to their stems and leaves; (2) Soil C-N-P densities significantly exceed biomass C-N-P densities; and (3) Soil factors predominantly determine the accumulation of C-N-P densities within the plant–soil system.

## 2. Results

### 2.1. Allocation of Biomass in Different Plant Components

**Figure 1:**
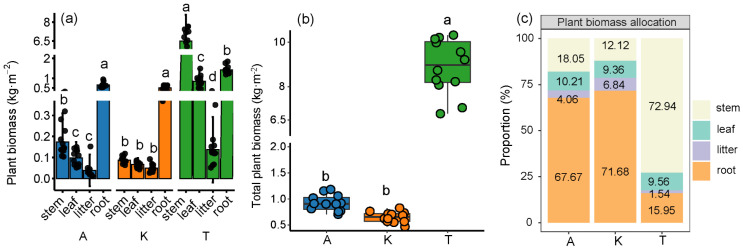
Allocation patterns and proportions of biomass in different components (**a**,**c**) and total biomass (**b**) of three different plants. A, *A. sparsifolia*; K, *K. caspia*; T, *T. ramosissima*. Different lowercase letters indicate significant differences among different components within the same plant (*p* < 0.05) (**a**). Different lowercase letters also indicate differences among different plants (*p* < 0.05) (**b**). Error bars are the standard error of the mean (n = 12).

In this study, the trend in total biomass of the three plant species was as follows: *T. ramosissima* > *A. sparsifolia* > *K. caspia*. The total biomass of *T. ramosissima* was 9.45 times and 13.46 times that of *A. sparsifolia* and *K. caspia*, respectively. The biomass distribution within *T. ramosissima* was ranked as follows: branches (72.94%) > roots (15.95%) > trunk (9.56%) > litter (1.54%). In *A. sparsifolia*, biomass was distributed as follows: roots (67.67%) > stems (18.05%) > leaves (10.21%) > litter (4.06%), while for *K. caspia*, the distribution was as follows: roots (71.68%) > stems (12.12%) > leaves (9.36%) > litter (6.84%) (Figure 1).

### 2.2. Carbon, Nitrogen, and Phosphorus Densities in Different Plant Components

**Figure 2:**
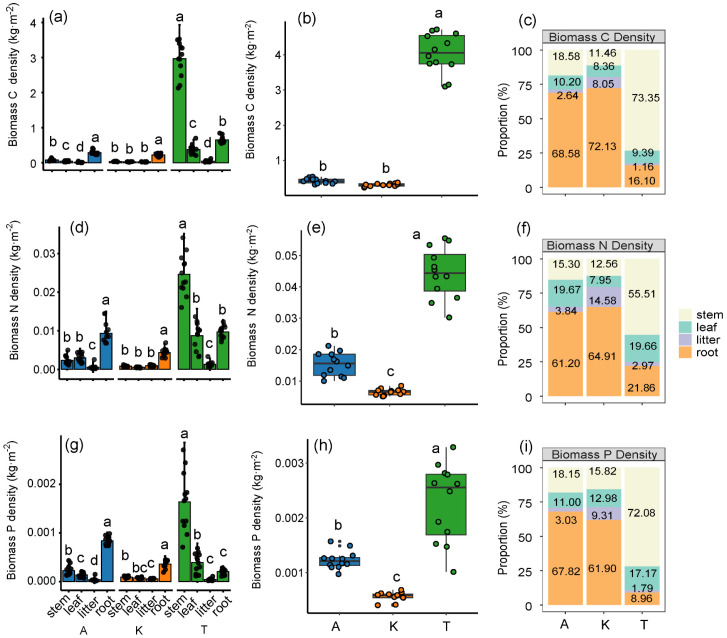
Absolute and relative distribution of biomass C-N-P densities of stem (trunk), leaf (branch), litter, and root under three desert plant species. A, *A. sparsifolia*; K, *K. caspia*; T, *T. ramosissima*. Biomass C-N-P densities are the sum of the stem (trunk), leaf (branch), litter and root (**a**,**d**,**g**). Different lowercase letters in the graphs represent differences between different components of the same plant (**a**,**c**,**d**,**f**,**g**,**i**) (*p* < 0.05), and different lowercase letters represent differences between total C-N-P densities of different plants (**b**,**e**,**h**) (*p* < 0.05). Error bars are the standard error of the mean.

The biomass C-N-P densities of *T. ramosissima* (C, 4.04 ± 0.56 kg m^−2^; N, 0.044 ± 0.008 kg m^−2^; P, 0.002 ± 0.0007 kg m^−2^) were significantly higher than those of *A. sparsifolia* and *K. caspia* (*p* < 0.05). Specifically, *T. ramosissima* recorded C-N-P densities that were 9.62 times higher for C, 2.93 times higher for N, and 2.00 times higher for P compared to *A. sparsifolia*, and 13.47 times, 6.29 times, and 4.00 times higher compared to *K. caspia*, respectively. The C-N-P densities in *T. ramosissima* biomass were predominantly influenced by the stem, while for *A. sparsifolia* and *K. caspia*, the roots were the main contributors (Figure 2). The variations in C-N-P densities in different components of *A. sparsifolia* and *K. caspia* followed the order: root > stem > leaf > litter. The biomass nutrient density trends were all consistently among three plants: C > N > P (Figure 2).

### 2.3. Distribution of Soil and Total C-N-P Densities

The order of soil nutrient densities was found to be C > P > N. No significant differences in soil organic carbon (SOC) density and soil TP density were observed among the three plants (*T. ramosissima*, *A. sparsifolia*, *K. caspia*) in the 0–200 cm soil layer; however, *A. sparsifolia* exhibited a soil TN density that was 0.09 kg m^−2^ higher than that of *T. ramosissima* (*p* < 0.05). The total C density variation among the three plants followed the order: *T. ramosissima* > *A. sparsifolia* > *K. caspia*, without significant differences in total N density and total P density among the plants. The total C density recorded for *A. sparsifolia*, *K. caspia*, and *T. ramosissima* were 6.21 ± 0.85 kg m^−2^, 6.18 ± 1.12 kg m^−2^, and 9.26 ± 0.99 kg m^−2^, respectively.

**Figure 3:**
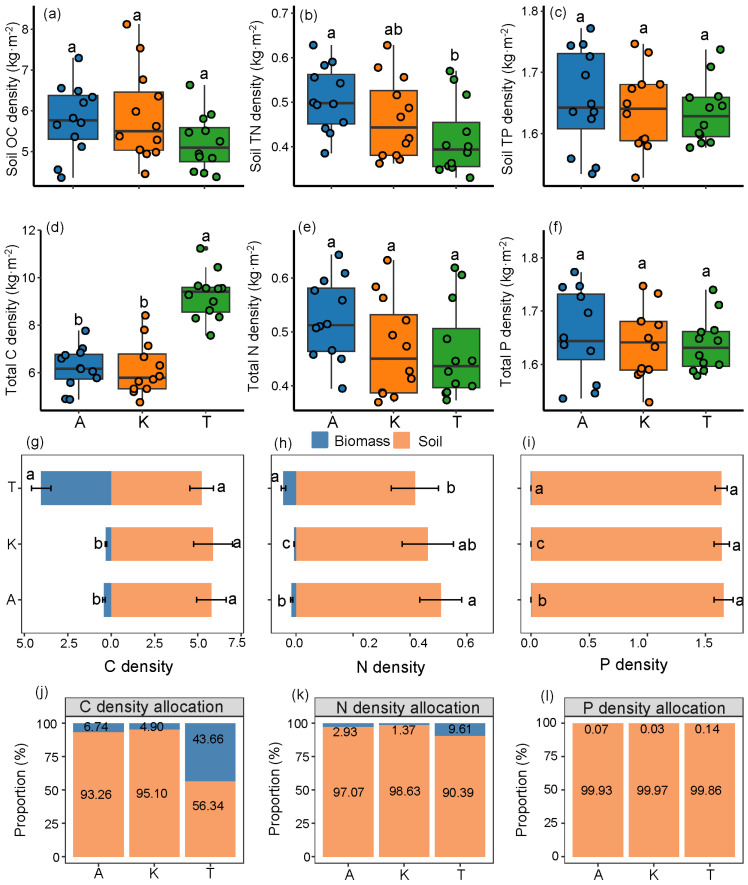
C-N-P densities characteristics under three desert plants: (**a**) SOC density, (**b**) soil TN density, (**c**) soil TP density, (**d**) total C density, (**e**) total N density, and (**f**) total P density. Different lowercase letters indicate significant differences among plant species (*p* < 0.05). Absolute distributions of C-N-P densities (**g**–**i**), and relative allocation (**j**–**l**) between biomass and soil pools. A, *A. sparsifolia*; K, *K. caspia*; T, *T. ramosissima*.

Soil C-N-P densities dominated the total nutrient pool accumulation, with soil C-N-P densities contributing between 56.34 and 95.10% of the total C density, 90.39 and 98.63% of the total N density, and 99.86 and 99.97% of the total P density, corresponding to the three plants. Specifically, for *A. sparsifolia*, the biomass C-N-P densities accounted for only 6.74%, 2.93%, and 0.07% of the total densities, respectively. For *K. caspia*, the biomass C-N-P densities contributed merely 4.90%, 1.37%, and 0.03%, while *T. Ramosissima* exhibited a biomass C density that represented 43.66% of the total C density. This finding indicates that the biomass C density of *T. ramosissima* is significantly greater than that of the herbaceous *A. sparsifolia* and *K. caspia* (Figure 3).

### 2.4. Correlation and Driving Factors of C-N-P Densities

**Figure 4:**
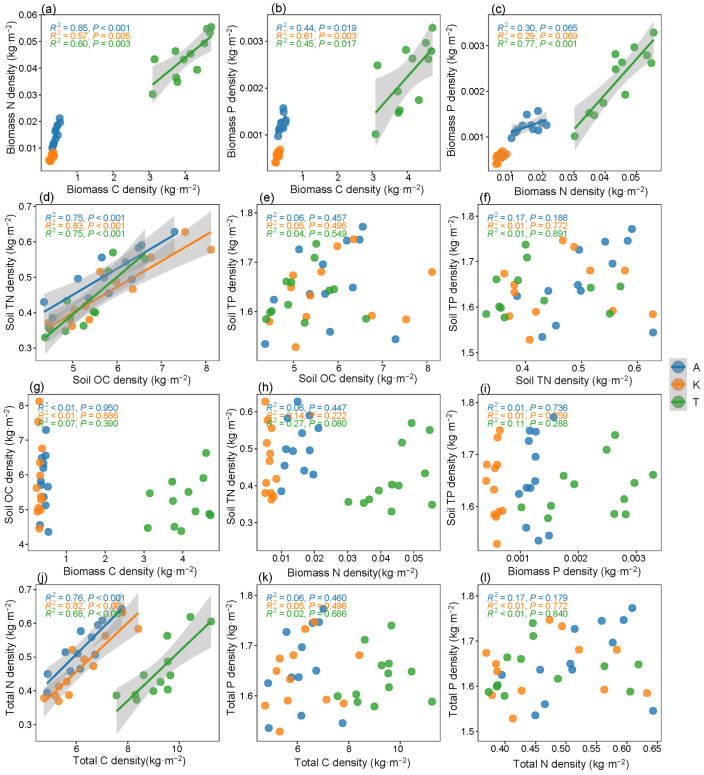
Regression relationships of C-N-P densities in corresponding biomass C-N-P densities (**a**–**c**), soil C-N-P densities (**d**–**f**), soil pools and biomass pools (**g**–**i**), total C-N-P densities (**j**–**l**). Red, green, and blue dots represent different indicators in A, *A. sparsifolia*; K, *K. caspia*; T, *T. ramosissima*, respectively. The solid lines were fitted by ordinary least-squares regressions, and the shadow areas corresponded to 95% confidence intervals.

Significant positive correlations were observed between biomass C density and biomass N density as well as between biomass C density and biomass P density across the three plant species. Similarly, SOC density and soil TN density exhibited significant positive correlations, highlighting a close relationship between C and N dynamics. Conversely, no significant correlations were identified between soil TP density and SOC density, between soil TN density and soil TP density, or between biomass N density and biomass P density, nor between total P density and total C or total N densities (Figure 4).

**Figure 5:**
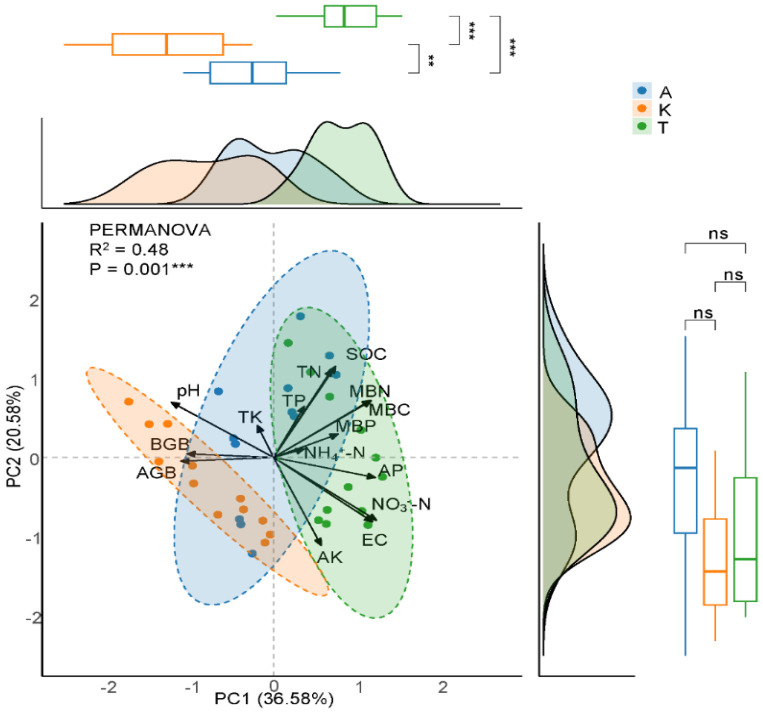
Principal component analysis (PCA) was used to identify the key dimensions of soil properties and plant biomass under three desert species. A, *A. sparsifolia*; K, *K. caspia*; T, *T. ramosissima*. Different colors represent the different plant species. We performed permutational multivariate analysis of variance (PERMANOVA) based on Bray–Curtis distances to test the statistical significance of soil and vegetation properties among the different plant species. Marginal distribution curves and box plots are shown alongside the PCA plot to illustrate the distribution and statistical differences between the PC1 and PC2 axes. ** and *** denote significant differences among the three plant species at *p* < 0.01 and 0.001 levels, respectively; ns indicates non-significant differences. AGB, aboveground biomass; BGB, 0–200 cm root biomass; TK, total potassium; TP, total phosphorus; TN, total nitrogen; SOC, soil organic carbon; MBC, microbial biomass carbon; MBN, microbial biomass nitrogen; MBP, microbial biomass phosphorus; NH_4_^+^-N, ammonium nitrogen; NO_3_^−^-N, nitrate nitrogen; AP, available phosphorus; EC, electrical conductivity; AK, available potassium.

The distribution of samples from different plant species was analyzed using principal component analysis (PCA), with the PC1 and PC2 axes collectively explaining 57.16% of the total variance (Figure 5). The first dimension (PC1) accounted for 36.58% of the variance and was mainly driven by soil physicochemical properties, such as NO_3_^−^-N, AP, EC, and MBN, while negative changes in PC1 correlated with AGB and pH levels. The second dimension (PC2) explained 20.58% of the variance, where SOC and pH positively affected the distribution, while AK had a negative effect. Among the three plants, samples of *K. caspia* were the most clustered, indicating minimal variation, while *A. Sparsifolia* displayed the greatest data dispersion. Although the distribution of all three species on the PC2 axis was relatively concentrated without significant differences, marked differences were observed on the PC1 axis (Figure 5).

**Figure 6:**
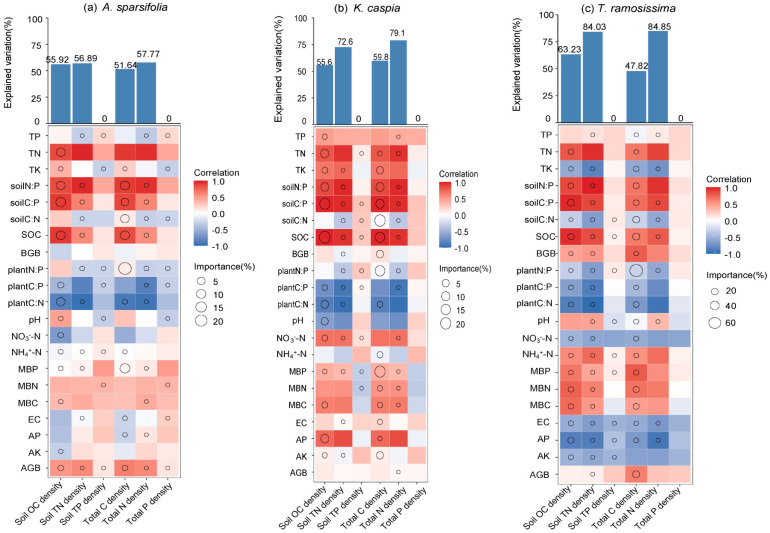
Importance of soil physicochemical properties, vegetation factors, and C-N-P stoichiometric ratios on SOC, soil TN, soil TP densities, total C-N-P densities. The percentage increase in mean squared error (MSE) is based on random forest analysis. The Spearman correlation between soil physicochemical properties (TN, TP, TK, SOC, NH_4_^+^-N, NO_3_^−^-N, AP, EC, AK), biotic factors (AGB, BGB), microbial biomass factors (MBC, MBN, MBP), and stoichiometric factors (plant C:N, C:P, N:P, soil C:N, C:P, and N:P ratios) with C-N-P densities is illustrated.

Soil TP density and total P density for the three plants were decoupled from any environmental factors (Figure 6). For *A. sparsifolia*, environmental factors explained 55.92%, 56.89%, 51.64%, and 57.77% of the variability in SOC density, soil TN density, total C density, and total N density, respectively. SOC density and total C density were primarily positively correlated with SOC, soil C:P ratios, soil N:P ratios, and AGB, while negatively correlated with plant C:N ratios, plant C:P ratios, EC, and AP. Similarly, soil TN density and total N density were positively correlated with AGB, TN, soil C:P ratios, and soil N:P ratios, and negatively correlated with plant C:N ratios and plant C:P ratios. For *K. caspia*, environmental factors accounted for 55.60%, 72.60%, 59.8%, and 79.1% of the variability in SOC density, soil TN density, total C density, and total N density, respectively. For *T. ramosissima*, environmental factors explained 63.23%, 84.03%, 47.82%, and 84.85% of the variability in SOC density, soil TN density, total C density, and total N density, respectively. In general, C and N densities were negatively correlated with TK, soil C:N ratios, plant C:N ratios, plant C:P ratios, plant N:P ratios, NO_3_^−^-N, EC, AP, and AK, while positively correlated with AGB, MBP, MBC, MBN, BGB, SOC, soil C: P, and soil N: P (Figure 6). In the desert ecosystem, the influence of soil microbial biomass factors on C and N densities is less significant compared to factors such as soil physicochemical properties.

## 3. Discussion

### 3.1. Allocation of C-N-P Densities Among Plants of Different Plant Life Forms

The total biomass of the shrub, *T. ramosissima*, is significantly greater than that of the herbaceous plants, *A. sparsifolia* and *K. caspia*. This biomass distribution pattern reflects evolutionary adaptations to xeric environments, influenced by phenotypic plasticity and species-specific functional traits characteristic of each species [35]. BGB (0–200 cm depth) for *A. sparsifolia* and *K. caspia* were 3.82–6.63 times and 5.94–7.60 times greater than the AGB, respectively (Figure 2), consistent with optimal partitioning theory [36]. This allocation pattern confirms the predominant belowground resource investment strategy of desert plants under nutrient and water limitations [37]. The development of extensive root systems is a critical adaptation for securing water and nutrient access, bolstering structural integrity that supports these vast root systems. Future studies should employ complete root system excavation coupled with isotopic tracing to fully characterize biomass allocation dynamics.

The C-N-P allocation strategies in different components of the three plant species were consistent with their biomass allocation patterns, where roots predominate in nutrient accumulation among desert herbaceous plants, confirming the first hypothesis. In contrast, *T. ramosissima* requires greater nitrogen and phosphorus investments in both its roots and trunks to support essential functions needed for photosynthesis. In *A. sparsifolia* and *K. caspia*, roots function as critical and metabolically active organs with higher C-N-P densities, facilitating protein synthesis, ATP production, nutrient uptake, root exudation, and symbiotic relationships with soil microorganisms [38]. Roots hold a competitive advantage in nutrient acquisition [39,40], which is supported by empirical evidence demonstrating their substantial contributions to total ecosystem C and N pools [41].

N and P densities substantially regulate C sequestration processes and ecosystem regulatory capacities, receiving significant attention in ecological research [42]. The biomass C-N-P densities of the three plants followed the pattern C density > N density > P density, with C density being markedly higher than N and P densities—an indication of carbon’s vital role in maintaining plant structural integrity [43] and reflecting efficient resource allocation adapted to arid environments [44]. Leguminous *A. sparsifolia* showed higher biomass N and P concentrations than non-leguminous *K. caspia* (Figure 2), reflecting its symbiotic nitrogen fixation capacity that enriches soil N pools and facilitates nutrient acquisition [45]. Additionally, leguminous roots often secrete phosphorus-mobilizing substances that increase P availability, leading to higher P content than that observed in non-leguminous plants [46]. These nutrient allocation strategies highlight the critical role of leguminous plants in nutrient cycling and soil enrichment in desert ecosystems.

### 3.2. Allocation of Total C-N-P Densities Among Plants of Different Life Forms

**Figure 7:**
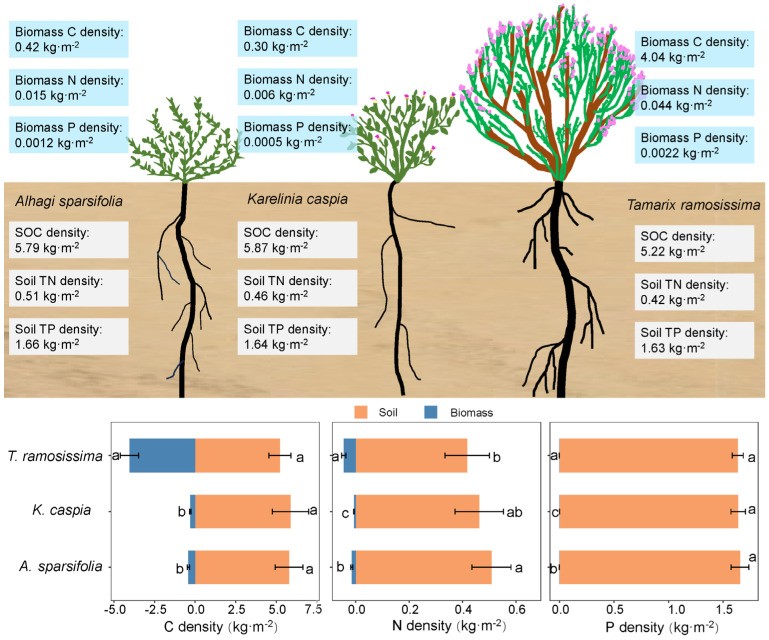
Conceptual framework illustrating differential C-N-P allocation in plant biomass and soil pools across three desert species. Different lowercase letters indicate significant differences among plant species (*p* < 0.05).

As predicted by our second hypothesis, the proportions of C-N-P densities in the soil (56.34–99.97%) were found to far exceed those in plant biomass (0.03–43.66%) (Figure 7). Comparatively, a study conducted in the Gurbantunggut Desert reported that the biomass C of *Haloxylon* + *Reaumuria* shrubs was 1.15 kg m^−2^, while SOC density at 0–5 m was 15.27 kg m^−2^ [47], similar to our findings, which suggested that both root C density and SOC density are significant contributors to C sequestration in this region. This reflects the fact that desert ecosystems have considerably higher belowground nutrient pools compared to other ecosystems, primarily due to reduced primary productivity following suppressed photosynthesis [48].

The total C-N-P densities of the three plant species followed the pattern C density > P density > N density (Figure 7), which aligns with certain findings in ecosystem studies [49,50]. The average soil C-N-P densities at the 0–200 cm depth for the three species were 5.63 kg m^−2^, 0.46 kg m^−2^, and 1.64 kg m^−2^, substantially lower than SOC density (13.16 kg m^−2^) and soil TN density (1.25 kg m^−2^) reported in China’s grasslands at the same depth [51], and below the average SOC density (12.27 kg m^−2^) in China’s forests (0–100 cm) [52]. However, given the extensive presence of desert ecosystems, their soil nutrient pools warrant attention and study.

This study indicated that there was no correlation between SOC density and soil TP density, nor was there a correlation between soil TN density and soil TP density. These observations mirror previous findings in temperate grasslands, which also reported a lack of correlation between SOC and soil TP density [53]. This decoupling of P from other factors can primarily be attributed to the stability of soil P, which is minimally affected by inputs from plants and litter. The uptake of phosphorus by plants is primarily determined by the metabolic demands for growth [54]. This may also be related to the P limitation in *A. sparsifolia* and *T. ramosissima*, and the N limitation in *K. caspia* [55]. Nutrient limitation, particularly N and P limitation, can exacerbate competition for resources between plants and soil microorganisms, further complicating the decoupling of C-P and N-P cycles in arid regions [56].

### 3.3. Drivers of Total C-N-P Densities Allocation

The random forest analysis results underscored that soil factors significantly influence the accumulation of SOC and soil TN densities, thereby confirming our third hypothesis. Soil C:P and N:P ratios had a more pronounced effect on the accumulation of C and N pools than microbial biomass and plant factors. As biomass accumulates, the inputs of nutrients from dead roots, litter, and root exudates enhance soil C and N densities [57,58]. Plant root systems exhibit various adaptations to enhance nutrient uptake, such as increased root branching, the development of root hairs, and mycorrhizal associations [2]. These adaptations enable plants to explore a larger soil volume and improve the efficiency of nutrient acquisition, particularly in environments with low nutrient availability [59]. For example, in nutrient-limited environments, plants with deeper and more extensive root systems can access nutrients from deeper soil layers, thus reducing competition with other plants and microorganisms [60].

Moreover, plants adjust their nutrient requirements in response to varying C:N:P ratios, influencing the release and mineralization of nutrients into the soil [61]. Plant stoichiometry metrics are negatively correlated with soil C-N-P densities (Figure 6), likely because higher plant C:N ratios (e.g., woody plant litter) decompose more slowly, leading in reduced N and P release [62]. The soil N:P ratio is generally lower than that of plants, indicating preferential P retention in plant biomass [63]. Soil C:N, C:P, and N:P ratios can significantly impact the abundance of functional genes involved in C and N cycling [64]. Soil physicochemical properties play a critical role in soil N pool accumulation, influenced by factors such as pH, soil structure, and moisture content, which directly affect N cycling [65]. Low soil nitrogen content reduces microbial activity, intensifying plant-microbe competition for nutrients and limiting primary productivity [66]. Biomass and nutrient allocation between aboveground and belowground components modulate the effects of soil stoichiometry on total C-N-P densities [67]. Furthermore, plants can influence soil C and N densities indirectly through their effects on substrate supply and soil mineralization [68].

In summary, the interactions among soil factors, vegetation factors, and stoichiometric ratios contribute to a complex regulatory network in this ecosystem. For example, N and P availability, plant nutrient uptake strategies, and soil microbial activity interact within this network, jointly regulating soil C and N accumulation [64]. When plant growth is constrained by nutrient availability, plants can adjust their C:N:P ratios and increase root exudation to stimulate microbial decomposition, thereby improving nutrient acquisition and growth [69]. Within this framework, even minor changes in a single factor can influence other factors, thereby altering soil C-N densities and the overall nutrient dynamics within this ecosystem. This study primarily focuses on local-scale investigations at the individual plant level. The distribution of C-N-P pools depends on plant life forms and interacting environmental factors. Unfortunately, this study, due to a smaller sample size, may have potential biases in the sampling process, focusing on a single time point and lacking consideration of other factors that may influence C-N-P allocation. We only selected three plants with similar sizes to control for ontogenetic effects but acknowledge that this approach may overlook variations in younger or older plants. Future studies should aim for more precise and diverse sampling, age-structured sampling to better assess how desert plants influence C-N-P pool allocation.

## 4. Materials and Methods

### 4.1. Study Area

**Figure 8:**
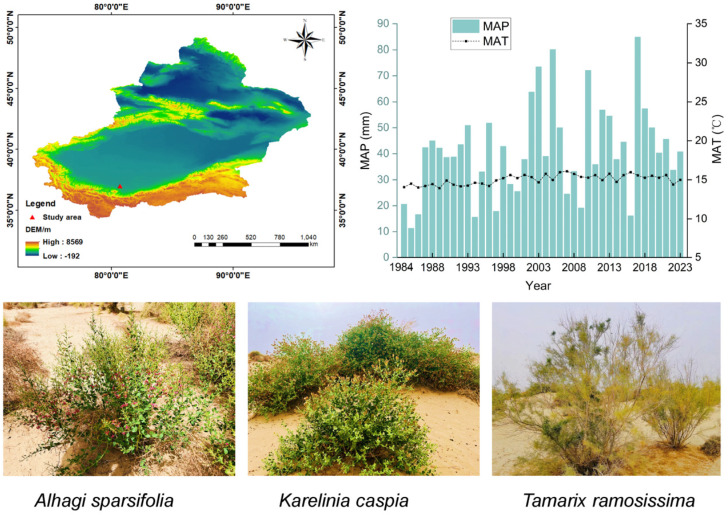
Study site location at the southern margin of China’s Taklamakan Desert, showing MAT and MAP data (1983–2023) from local meteorological stations. Featured species: two desert herbs (*A. sparsifolia*, *K. caspia*) and one desert shrub (*T. ramosissima*).

The experiments were conducted in a native habitat desert near the Cele Desert Research Station, Chinese Academy of Sciences (80°43′45″ E, 37°00′57″ N), located on the southern edge of the Taklamakan Desert (Figure 8). The mean annual precipitation (MAP), mean annual temperature (MAT), and annual potential evaporation are 41.45 mm, 15.04 °C, and 2956 mm, respectively. This region is predominantly affected by northwest winds, characterized by an extremely arid climate, low soil organic matter (SOM) content, and frequent occurrences of wind erosion. The soils in the research sites are classified as Arenosols, known for their poor water retention capacity, which makes them prone to nutrient leaching. After extensive habitat adaptation, only a limited number of drought- and sand-tolerant shrubs and herbaceous plants have managed to thrive in the desert–oasis transition zone. The dominant natural species observed include *A. sparsifolia*, *K. caspia*, and *T. ramosissima*, along with *Phragmites australis* (Cav.) Trin. ex Steud. The growth of these perennial desert plants primarily relies on their extensive root systems to access groundwater, supplemented by meltwater from snowfall during July and August. These species play a crucial role in maintaining ecological stability and mitigating soil erosion in this region.

### 4.2. Experimental Design and Samples Collocation

We surveyed distinct communities of *A. sparsifolia*, *K. caspia*, and *T. ramosissima* in the desert-oasis transition zone of Cele County during July and August 2023. *A. sparsifolia* (a legume), and *K. caspia* (non-legume) are perennial herbaceous plants, while *T. ramosissima* was classified as a perennial shrub. Using dendrochronology, we selected *T. ramosissima* samples of consistent age for the study. To minimize the high heterogeneity of the study area, isolated single-species plots (three plots of 10 m × 10 m) with similar plant heights and canopy diameters were chosen. The three sites are located within a 300 m radius, are characterized by similar slope gradients, soil textures, and altitudes, and are devoid of anthropogenic influences such as mowing and grazing. Initially, we recorded the height and canopy diameter of all plants in the plots and selected four well-growing individuals as standard plants (3 × 4 = 12 replicates). All selected plants were in a stable growth phase and considered representative.

To obtain the aboveground biomass (AGB) of each plant, we individually collected and separated the entire stem, leaf, and 0–200 cm root system of standardized plants in situ across all three plots, resulting in a total of 144 plant samples. These specimens were subsequently transported to the laboratory, where they were dried in an oven at 75 °C for 96 to 192 h reaching a constant weight, allowing for the determination of C, N, and P concentrations. For the collection of the 0–200 cm root system, manual shovels were used to excavate the soil. Initially, a 2 m × 2 m × 2 m pit was dug and the coarse roots were carefully cleaned with water and a brush to retrieve the complete root system from the 0–200 cm soil depth, which was then brought back to the laboratory. In this study, the AGB of *A. sparsifolia* and *K. caspia* was separated into stems and leaves, while the AGB of *T. ramosissima* was divided into stem (trunks) and leaves (assimilative branches). Additionally, litter corresponding to each plant was collected from an area measuring 1 m × 1 m, dried to a constant weight, and measured for dry matter weight. Due to the potential mass loss from cutting and weighing the entire 0–200 cm root system, we chose to air-dry the roots of the herbaceous plants and the branches of *T. ramosissima* in the laboratory for a period of 30 days before measuring their dry matter weight. The root systems of these three plants primarily consisted of main and coarse roots, with negligible quantities of fine roots. Despite the limited capture of fine roots, we believe the biomass data are trustworthy. For the collection of deeper belowground parts, we acknowledge that many roots of these three plants are distributed at depths greater than the 200 cm depth. Thus, we lost some roots, but this work was laborious and challenging. After collecting plant root biomass, we also collected soil samples at depths of 0–20, 20–60, 60–100, 100–150, and 150–200 cm. After mixing soil samples from five different depths (3 plants × 12 replicates = 36 total soil samples), they were divided into two portions. One portion was air-dried indoors, passed through a 2 mm sieve, and measured for soil physicochemical properties; the other portion was stored at −4 °C for soil microbial biomass.

### 4.3. Plant C-N-P Analysis

After collecting samples of different plant components, they were ground using a ball mill and passed through a 0.15 mm sieve. The concentrations of C, N, and P in different plant components (stems, leaves, litter, roots) were determined using an inductively coupled plasma atomic absorption spectrometer (ICP-ABS Hitachi Z-5000, Chiyoda, Japan). The main steps for measurement included washing the plant samples to remove impurities, drying them to a constant weight, grinding, and digesting with nitric acid, hydrochloric acid, and hydrogen peroxide. The digested samples were then diluted and analyzed using the ICP-ABS instrument to determine the concentrations of the elements.

### 4.4. Soil Physicochemical and Microbial Biomass Analysis

Soil bulk density was measured using the cutting ring method (volume of 100 cm^3^), and the average value across different depths was considered as the soil bulk density for the 0–200 cm layer. Soil organic carbon (SOC) content was determined using the K_2_Cr_2_O_7_-H_2_SO_4_ oxidation method, total nitrogen (TN) was measured using the Kjeldahl method, total phosphorus (TP) was determined via the sodium hydroxide fusion–molybdenum antimony colorimetric method, and total potassium (TK) was assessed with atomic absorption spectrophotometry. Available phosphorus (AP) was determined using the sodium bicarbonate extraction-molybdenum antimony colorimetric method, while available potassium (AK) was measured using the ammonium acetate extraction-flame photometer method. Nitrate nitrogen (NO_3_^−^-N) and ammonium nitrogen (NH_4_^+^-N) were quantified by spectrophotometric analysis. Soil electrical conductivity (EC) and pH levels were determined using soil and distilled water mixtures at ratios of 1:5 (*w*/*v*) and 1:2.5 (*w*/*v*), respectively. Microbial biomass carbon (MBC), microbial biomass nitrogen (MBN), and microbial biomass phosphorus (MBP) were assessed using the chloroform fumigation-extraction method [70]. Additionally, the average concentrations of C, N, and P in different plant components were used to calculate plant C, N, and P, along with the corresponding plant and soil C:N, C:P, and N:P ratios for further analysis of their influence on total C-N-P densities.

### 4.5. Data Calculations

Biomass C-N-P densities were computed using the following equation [71]:

(1) $$\mathrm{Biomass}\;X\;\mathrm{density}=\frac{X_{p\;}\times \;M_{p}}{1000}$$

where biomass X density, is the biomass C density, N density, or P density (kg m^−2^). It includes leaf, stem, aboveground litter, and root C, N, or P density; X_p_ is the C, N, or P concentration of different components (g kg^−1^); M_p_, is the biomass of the different components (kg m^−2^). Total biomass is the sum of the biomass of different components. C, N, and P densities are the sum of the C, N, and P densities of different components.

Biomass C, N, and P densities were computed using the following equation [72]:

(2) $$\mathrm{Soil}\;X\;\mathrm{density}=\frac{X_{i}\;\times \;D\;\times \;\mathrm{BD}}{100}$$

where X is SOC density, TN density, TP density (kg m^−2^), X_i_ is SOC, soil TN, soil TP content at 0–200 cm (g kg^−1^), BD is the bulk soil density (g cm^−3^); and D is the soil depth (200 cm). We define the total C, N, P density as the sum of biomass C, N, P density and soil C, N, P density.

(3) Total X density=biomass X density+soil X density

where X is C density, N density, and P density (kg m^−2^).

### 4.6. Statistical Analysis

One-way ANOVA was conducted to examine the differences in different plant organ components and total biomass, as well as the biomass C-N-P densities, soil C-N-P densities, and total C-N-P densities among the three species. Before performing the ANOVA analysis, the homogeneity of the variance was evaluated (Kolmogorov–Smirnov test) and data underwent log-transformation if the assumption of homogeneity was violated. Correlations between biomass, soil, as well as total C-N-P densities, were analyzed using the “ggplot2” package. The PERMANOVA analysis with 999 permutations using the “vegan” package was performed to test the significant differences in PCA eigenvalues among different plants. Spearman’s correlation analysis was used to examine the relationships between soil physicochemical properties, microbial biomass, stoichiometry, and soil C-N-P densities, as well as total C-N-P densities. The random forest analysis with 1234 trees and 1000 permutations (“rfPermute” package) was conducted to identify the important driving factors and total explanatory power that influence soil and total C-N-P densities, and the significance of these factors and the random forest model was assessed by the percentage increase in mean squared error. All these analyses and plots were performed using R v.4.4.2.

## 5. Conclusions

This study comprehensively evaluated the biomass and soil C-N-P densities of three desert plants in a hyper-arid desert environment. The results show that the biomass of the shrub *T. ramosissima* is significantly greater than that of the herbaceous plants *A. sparsifolia* and *K. caspia*. For both *A. sparsifolia* and *K. caspia*, biomass allocation is primarily directed toward roots, followed by stems and leaves, with the least allocation to litter. In contrast, *T. ramosissima* allocates its biomass in the order of trunks > roots > branches > litter. The C-N-P densities in plants align with their biomass allocation strategies, with the sequence of densities being *T. ramosissima* > *A. sparsifolia* > *K. caspia*. While differences in soil C-P densities among plant species were not significant, the soil N density in association with leguminous *A. sparsifolia* was significantly higher than that of *K. caspia* and *T. ramosissima*. Overall, the soil C-N-P pools dominate the C-N-P densities in the plant–soil system. The allocation of total nutrient pools consistently follows the pattern of C density > N density > P density, with the sequence of changes being *T. ramosissima* > *A. sparsifolia* > *K. caspia*. The processes underlying C and N accumulation in the plant–soil system of desert plants are primarily driven by soil factors rather than by vegetation factors. Notably, total P density showed no correlation with environmental variables or total C and N densities. Additionally, the impact of soil microbial biomass appeared less significant when compared to that of soil physicochemical properties. This study elucidates nutrient allocation strategies in desert plants, yet further investigations are required to unravel the mechanistic drivers of C-N-P densities across diverse desert plant species.

## Data Availability

The original contributions presented in this study are included in the article. Further inquiries can be directed to the corresponding authors.
